# Participation in Mass Gatherings Can Benefit Well-Being: Longitudinal and Control Data from a North Indian Hindu Pilgrimage Event

**DOI:** 10.1371/journal.pone.0047291

**Published:** 2012-10-17

**Authors:** Shruti Tewari, Sammyh Khan, Nick Hopkins, Narayanan Srinivasan, Stephen Reicher

**Affiliations:** 1 Centre of Behavioural and Cognitive Sciences, University of Allahabad, Allahabad, Uttar Pradesh, India; 2 School of Psychology, University of Dundee, Dundee, Scotland, United Kingdom; 3 School of Psychology, University of St Andrews, St Andrews, Scotland, United Kingdom; Umeå University, Sweden

## Abstract

How does participation in a long-duration mass gathering (such as a pilgrimage event) impact well-being? There are good reasons to believe such collective events pose risks to health. There are risks associated with communicable diseases. Moreover, the physical conditions at such events (noise, crowding, harsh conditions) are often detrimental to well-being. Yet, at the same time, social psychological research suggests participation in group-related activities can impact well-being positively, and we therefore investigated if participating in a long-duration mass gathering can actually bring such benefits. In our research we studied one of the world's largest collective events – a demanding month-long Hindu religious festival in North India. Participants (comprising 416 pilgrims who attended the gathering for the whole month of its duration, and 127 controls who did not) completed measures of self-assessed well-being and symptoms of ill-health at two time points. The first was a month before the gathering commenced, the second was a month after it finished. We found that those participating in this collective event reported a longitudinal increase in well-being relative to those who did not participate. Our data therefore imply we should reconceptualise how mass gatherings impact individuals. Although such gatherings can entail significant health risks, the benefits for well-being also need recognition. Indeed, an exclusive focus on risk is misleading and limits our understanding of why such events may be so attractive. More importantly, as our research is longitudinal and includes a control group, our work adds robust evidence to the social psychological literature concerning the relationship between participation in social group activities and well-being.

## Introduction

There is an increasing recognition that our well-being is shaped by social factors such as our social relationships [1]–[3]. Various processes have been posited to explain this relationship. Some of these concern the beliefs and practices of particular groups. Others concern the more general features of group life.

For example, being religious has a positive impact on well-being. In part, this is because religious beliefs provide cognitive schemas or encourage meditative practices relevant to the appraisal of life events and coping [4], [5]. In part, it is also because of one's participation in a congregation and the fact that one acts alongside others [6]–[11]. In similar vein, research on work groups operating in stressful environments shows that the more one feels a part of the team, the better one's well-being [12], [13], and evidence from studies of the elderly living in residential homes reveals that a range of activities (from drinking water to avoid dehydration to reminiscing about the past) bring about benefits because they are done in groups [14], [15].

The underlying psychological process that link group membership with well-being is argued to derive from a sense of shared identity (‘we-ness’) that develops in groups. This leads people to experience mutual trust, respect and cooperation [16], [17]. It can also lead people to expect support from their fellow group members and develop greater resilience [18]. Building upon such insights it has been argued that since we spend most of our time in the company of others, we should study health in group settings [19].

To date, though, research in this tradition has been done principally upon small and organized groups. Our focus here is on a very different type of collectivity – a long-duration mass gathering. When it comes to such gatherings (e.g., pilgrimage events such as the *Hajj*) a very different viewpoint tends to prevail. They are viewed as posing significant risks to well-being and health [20], [21]. Some risks concern the dangers posed by communicable diseases [22]. Some relate to the rudimentary living and sanitary facilities at such events [23]. Still other risks arise from the presence of others. These latter include the dangers of crushing and the stresses of living in crowded and noisy conditions. For example, the dense crowding characteristic of collective events can increase stress and blood pressure [24]. So too, the loud noise levels that characterise mass gatherings, can increase stress and mental health symptoms [25].

However, an exclusive focus on the risks of participation may lead us to overlook the potential benefits that participation in mass gatherings can have. Although not specifically focussed on well-being, social anthropological theory has long argued that mass gatherings (e.g., carnivals and religious festivals) can be joyous occasions and involve a sense of intimacy even between people who do not know each other [26], [27]. Moreover, such theory has spoken of the ways in which mass gatherings revivify social bonds and re-establish group identities. However, as several researchers lament, these more positive features of collective events are routinely overlooked in much contemporary academic research [28]. Indeed as Getz shows, even researchers interested in the tourism associated with mass events (e.g., sports events, cultural festivals, etc.) tend to overlook how participation may impact positively on participants' well-being [29], [30]. Instead, such research typically focuses on the economic and environmental impacts of these events or on issues of safety management.

In the research reported here we seek to rebalance the situation by addressing the neglected benefits of collective participation. Specifically we investigate how participation in one of the world's largest collective events - the *Magh Mela* at Allahabad in Northern India – impacts participants' well-being [31]. Our data are quantitative and derived from an orally-administered questionnaire concerning well-being. These self-report data were obtained before and after the collective event, and from a sample attending the event and from a sample of controls (comparable others who did not attend). Before elaborating on our design and measures, we describe the context and nature of the event more fully.

### Setting

Every year, in the Hindu month of *Magh* (mid-January to mid-February), pilgrims gather at the confluence of the Ganges and Yamuna rivers to perform a series of sacred rituals - notably to bathe in the rivers. The event is on a 12-year cycle. In the twelfth year (the *Maha Kumbh Mela*) it is claimed that up to 50 million people attend and over 10 million can be present on a single bathing day. Every six years (the *Ardh Kumbh Mela*), somewhere in the region of 20 million participate. Yet, even for the ‘routine’ yearly gatherings – the *Magh Mela* - millions attend, and hundreds of thousands undertake to remain for the full month.

Those pilgrims who stay for the whole month (known as *Kalpwasis*) live in conditions that are more difficult than those they experience at home. They live in rudimentary tents without heating, often without sanitary facilities, sleeping on the ground and experiencing night-time temperatures approaching zero centigrade. The event is also very crowded, and again this contrasts with life in the villages from which the pilgrims come. The crowds make walking to the bathing areas difficult and on days that bathing is judged particularly auspicious in terms of Hindu traditions, it can take several hours to walk a kilometre or so. Another striking feature of the event is its noise level. A vast array of competing loudspeakers broadcast religious discourses, songs, announcements and other administrative information throughout the day (and into the night). Our measurements show that on an ordinary day the noise level rarely falls below 75 dB and is often 80–85 dB. It is noteworthy that, according to the US National Institute on Deafness and Other Communication Disorders, exposure to 85 dB or more will cause hearing damage after 8 hours. Again, the contrast between this aspect of the environment and that of the pilgrims' home villages is striking. All in all, life in the Mela is difficult and demanding.

Even if enduring hardship is integral to the act of pilgrimage [32] and even if such hardships do not deter pilgrims from attending, these various circumstances - unsanitary conditions, severe cold, dense crowding and intense noise - are all those that would be expected to be bad for well-being. But are they? We ask if, in the light of social psychological research identifying associations between involvement in social group-related activities and well-being, participation in this mass gathering could impact well-being positively. To find that participation in such an arduous mass gathering impacts well-being positively would be striking, and would underline the wider relevance and applicability of psychological research concerning the benefits associated with social participation in group activities.

## Methods

We recruited a sample of those who participated in the Magh Mela for the full month-long festival (Kalpwasis) and a sample of comparable others who did not attend at all (Controls), and asked both about their well-being and their experience of various symptoms of ill-health. We gathered these data one month before the 2011 Magh Mela (Time 1 - T1) and one month after it had ended (Time 2 - T2), and explored the degree to which the Kalpwasis sample showed a longitudinal increase in reported well-being compared to the Controls.

### Participants

The sample consisted of 543 respondents providing data at two time points: before (T1) and after (T2) the Mela. Of these, 416 were Kalpwasis who attended the Mela and 127 Control others who did not. In the first round of data collection (pre-Mela), the sample comprised a total of 792 respondents (604 planning to attend the Mela and 188 not). With attrition, 249 (31.44%) participants were lost giving an overall completion rate of 68.56%. Attrition was equivalent amongst those planning to attend (188 or 31.13%) and the controls (61 or 32.45%). Analyses of the pre-mela data show no differences in the socio-demographic attributes (age, gender, marital-status, educational level, and caste) of our final sample and those lost through attrition.

The final samples of Kalpwasis and Controls were comparable in their socio-demographic attributes: Age (Kalpwasis *M* = 64.38 years, *SD* = 9.32; Controls *M* = 60.90, *SD* = 13.44 years); Gender (Kalpwasis: 57.0% female; Controls 50.4% female); Caste (Kalpwasis: 92.3% *General Caste*, 7.7% *Other Backward Caste* ; Controls: 85.8% *General Caste*, 14.2% *Other Backward Caste*). In all analyses involving comparisons between Kalpwasis and Controls, age, gender, caste, marital and educational-status were employed as covariates.

### Ethics statement

This study was approved by the Ethics Committees of the University of Dundee and the University of Allahabad. When approaching potential participants, the researchers gave an overview of the questions to be asked. Participants gave informed consent to participate. As many were unable to read and write, this consent was oral. The decision to seek oral rather than written consent was approved by the above Ethics Committees. The procedure for documenting that oral consent was given was as follows. After explaining the research, participants were asked a formal consent question: ‘Do we have your consent to participate in this survey study?’ The researcher registered the answer as ‘Yes’ or ‘No’ on the response sheet. The researcher also signed their name on this response sheet.

### Procedure

**Figure 1:**
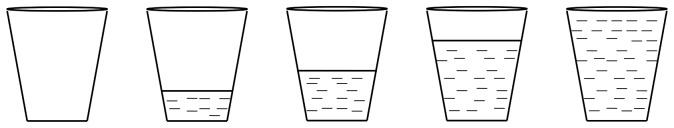
Visual representation of 5-point scale employing glasses with varying levels of water. The anchoring of the empty and full glasses varied according to the questions asked (see text).

Data were gathered with a questionnaire administered orally in Hindi by a trained team of 10 Hindi-speaking field investigators at participants' homes which were within a radius of 100–120 KMs from Allahabad, India. As a sample from rural India has little (if any) experience of questionnaire surveys, and still less experience of using 5-point scales, we took considerable care to conduct the research in a manner that was intelligible. For example, to convey the concept of a 5-point scale, we showed participants drawings of five glasses containing increasing levels of water (ranging from empty to full) and used these to explain to participants how they could communicate their level of well-being and the degree to which they experienced symptoms of ill-health (see Figure 1). The survey took approximately 30 minutes to complete. To ensure the translation was conceptually appropriate, the questionnaire was translated and back-translated (English-Hindi-English) by two independent groups of translators. Any differences between the translations were resolved by improving the questionnaire items. Before the survey was administered, the scales were piloted amongst both illiterate and literate Hindi-speaking participants. This ensured the items were clear and intelligible to participants of widely varying educational backgrounds.

The T1 survey was administered between December 1^st^ and 15^th^, 2010 - one month before the beginning of the 2011 Magh Mela. The T2 survey was administered between March 3^rd^ and 15^th^, 2011 - one month after the Mela's conclusion. The average time difference between T1 and T2 was 90 days (*SD* = 3 days). An independent samples *t*-test revealed no significant difference between the Kalpwasis and Controls in the number of days between T1 and T2, *t*(541) = 1.23, *p* = .20, *Cohen's d* = .11.

### Measures

#### Well-being

Reports of well-being were obtained with three items from the core module of the Centers for Disease Control and Prevention Health Related Quality of Life Measure (CDC HRQOL-14) [33]: “*Over the last week, how would you describe your physical health”,* “*Over the last week, how would you describe your state of mind”,* “*Over the last week, how would you describe your energy levels.”* Responses were gathered on a 5-point scale illustrated with glasses containing varying levels of water. The empty glass (scored ‘1’) was anchored, “*Very Poor*”, the full glass (scored ‘5’) was anchored, “*Very Good*”. Item scores were averaged together such that a higher score indicated better well-being. The reliability (Cronbach's alpha) of this scale was excellent (T1 Participants = .77; T2 Participants = .86; T1 Controls = .81; T2 Controls = .91).

#### Symptoms of Ill-health

Participants' reported well-being was also measured with six items concerning symptoms of ill-health taken from a scale specifically developed for use in the Indian Subcontinent [34], [35]. Three items concerned psychological well-being: “*Over the last week, to what extent have you felt anxious without any reason”,* “*Over the last week, to what extent have you felt restless without any reason”,* “*Over the last week, to what extent have you felt irritable without any reason?*” Three concerned physical well-being: “*Over the last week, to what extent have you suffered from body-aches and pains”,* “*Over the last week, to what extent have you suffered from breathlessness”,* “*Over the last week, to what extent have you suffered from headaches?*” Again responses were gathered on a 5-point scale illustrated with glasses containing varying levels of water and anchored: 1="*Not at all*”, 5="*A lot*” (in Hindi the word used translates literally as “*Completely*”). Item scores were averaged together such that a lower score indicated less symptoms of ill-health. The reliability (Cronbach's alpha) of this scale was excellent (T1 Participants = .81; T2 Participants = .85; T1 Controls = .84; T2 Controls = .84).

## Results

Average levels of self-assessed Well-being and Symptoms of ill-health are reported for Kalpwasis and Controls before and after the Mela in Table 1.

**Table 1:** Well-being and Symptoms of Ill-health amongst Kalpwasis and Controls at T1 and T2.

		Kalpwasis		Controls	
		T1	T2	T1	T2
**Well-being**	EMM	3.35	3.62	3.30	3.30
	SE	.04	.04	.08	.08
**Symptoms of ill-health**	EMM	2.04	1.66	2.16	1.96
	SE	.04	.04	.07	.07

Estimated Marginal Means (EMM) and Standard Errors (SE).

### Well-being

**Figure 2:**
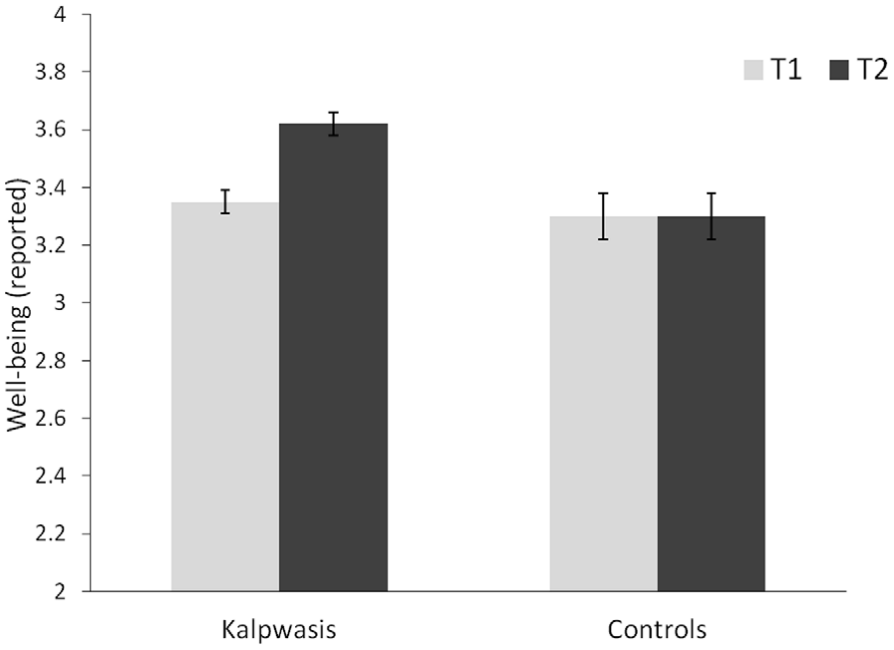
Well-being amongst Kalpwasis and Controls at T1 and T2. Estimated Marginal Means and Standard Errors.

Participants' levels of Well-being were inspected in a 2 (Condition: Kalpwasis/Controls)×2 (Time: T1/T2) Mixed Factorial ANCOVA (in which age, gender, caste, marital and educational-status featured as covariates). This showed no effect of Time, *F*(1, 533) = .01, *p* = .93, *η_p_^2^*<.001, and that Kalpwasis reported better well-being than Control participants, *F* (1,533) = 6.05, *p* = .014, *η_p_^2^* = .011. However, and most importantly, interpretation of this effect was qualified by an interaction, F (1, 533) = 6.23, p = .013, *η_p_^2^* = .012. The relevant Estimated Marginal Means and Standard Errors are plotted in Figure 2. Decomposing this interaction shows that whereas there was no difference in well-being between Kalpwasis and Controls at T1 (Kalpwasis *EMM = *3.35, *SE = *.04; Controls *EMM = *3.30, *SE* = .08), *F* (1, 533) = .40, *p* = .53, *η_p_^2^* = .001, at T2 Kalpwasis reported better Well-being (*EM = *3.62, *SE = *.04) than Controls (*EMM* = 3.30, *SE* = .08), *F*(1, 533) = 11.71, *p* = .001, *η_p_^2^* = .021. Moreover, inspecting the Kalpwasis' data revealed an improvement in Well-being from T1 (*EMM* = 3.35, *SE* = 04) to T2 (*EMM* = 3.62, *SE* = .04), *F* (1, 415) = 31.25, *p*<·001, *η_p_^2^* = .07. In contrast, there was no such improvement amongst Controls (T1: *EMM* = 3.30; *SE* = .08; T2 *EMM = *3.30, *SE* = .08), *F* (1, 126) = .001, *p* = .98, *η_p_^2^*<.001.

### Symptoms of ill-health

**Figure 3:**
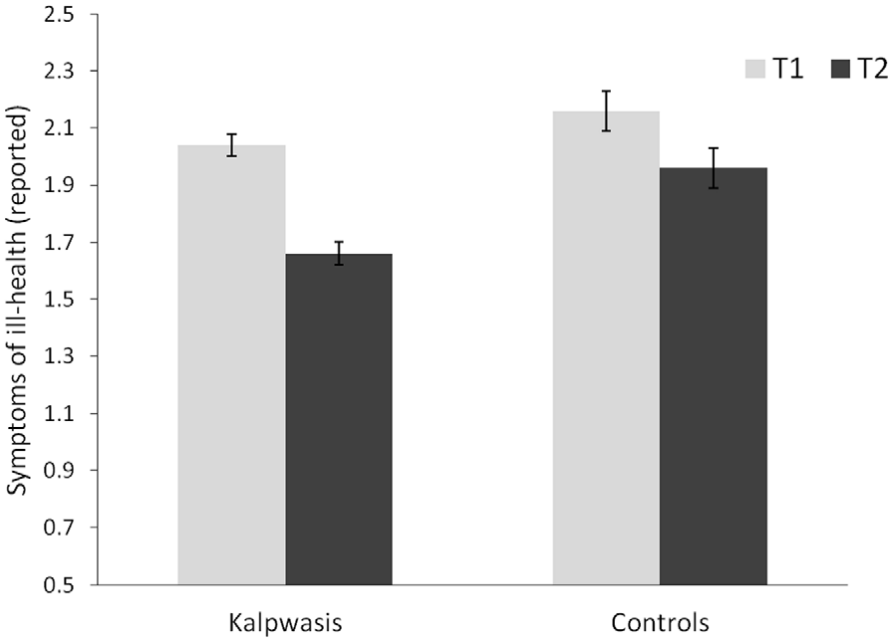
Symptoms of Ill-health amongst Kalpwasis and Controls at T1 and T2. Estimated Marginal Means and Standard Errors.

Participants' reported symptoms of ill-health were inspected in a similar 2 (Condition: Kalpwasis/Controls)×2 (Time: T1/T2) Mixed Factorial ANCOVA (with age, gender, caste, marital and educational-status featuring as covariates). This showed a similar pattern. There was no main effect of Time, *F*(1, 533) = 1.48, *p* = .23, *η_p_^2^* = .003, and Kalpwasis reported less symptoms than Controls, *F* (1,533) = 9.45, *p* = .002, *η_p_^2^* = .017. However, interpretation of this main effect was again qualified by the predicted interaction, *F* (1, 533) = 4.23, *p* = .04, *η_p_^2^* = .008. The relevant Estimated Marginal Means and Standard Errors are plotted in Figure 3. Decomposing this interaction shows that whereas there was no difference between the two groups at T1 (Kalpwasis *EMM = *2.04, *SE = *.04; Controls *EMM* = 2.16, *SE* = .07), *F* (1, 533) = 2.03, *p* = .16, *η_p_^2^* = .004, at T2 Kalpwasis reported significantly fewer symptoms (*EMM = *1.66, *SD = *.04) than Controls (*EMM* = 1.96, *SE* = .07), *F* (1, 533) = 15.02, *p*<.001, *η_p_^2^* = .027. Moreover, for the Kalpwasis there was a sharper decrease in their reporting of symptoms from T1 (*EMM* = 2.04, *SE* = .04) to T2 (*EMM* = 1.66, *SE* = .04), *F* (1, 415) = 88.35, *p*<.001, *η_p_^2^* = .176, than amongst the Controls (T1 *EMM* = 2.16, *SE* = .07; T2 *EMM* = 1.96, *SE* = .07), *F*(1, 126) = 6.67, *p* = .011, *η_p_^2^* = .05.

## Discussion

Our results reveal that whilst Kalpwasis and Controls had comparable pre-Mela scores on both of our measures (Well-being and Symptoms of Ill-health), their post-Mela scores diverge to show the Kalpwasis doing better. With regards to the Well-being measure, the Kalpwasis showed an improvement from before to after the Mela whereas the Controls did not. With regards to their reporting of Symptoms of Ill-health, the pattern is similar. Whilst there is some evidence that both groups improved (perhaps because the first measure was taken in the winter and the second was taken in spring) the improvement was greater for the Kalpwasis.

Based on the findings obtained with these two measures we have good grounds for believing that taking part in this demanding collective event did indeed have beneficial effects. These are all the more striking for the fact that we set out to study a crowded, noisy and physically-testing mass gathering.

The next obvious question is why, and hence when, participation may result in such outcomes. Being able to follow one's religious beliefs and enact religious rituals is likely to be identity-affirming and this may contribute to a sense of well-being. Moreover, the collective nature of this enactment is also likely to be important [36]. As discussed in the introduction, there is good evidence that a key ingredient in the association between well-being and religious belief/practice concerns the collective dimension to religious activity. This underlines the importance of considering how participation in group activities can be beneficial through leading people to feel supported by others and hence better able to control their everyday lives.

Our findings require that we reappraise the way we look at mass gatherings. Such gatherings are judged as posing a variety of risks. Certainly these risks are serious and should not be underplayed: planning for the control of disease and the through-flow of people at such events is important. However, we also need to consider the benefits associated with such events. Such benefits may be diverse [29], [30]. Here we show they can include well-being. Moreover, we found such benefits obtained even where the physical conditions are harsh (as they are in the collective event studied here). Recognizing the potential for such benefits is important. They help explain some of the attractions of such events and hence why people may be so determined to participate (of obvious importance for event-management). More generally our data provide distinctive evidence for the idea that participation in the social life of a group membership impacts well-being positively. As much work addressing the relationship between social group processes and well-being is cross-sectional in nature (rather than longitudinal) and derived from research conducted in Europe and North America, our data are particularly significant.
